# Quality of life and the impact of drug toxicities in a South African community-based antiretroviral programme

**DOI:** 10.1186/1758-2652-12-5

**Published:** 2009-04-24

**Authors:** Jennifer Pitt, Landon Myer, Robin Wood

**Affiliations:** 1 Desmond Tutu HIV Foundation, Institute of Infectious Diseases and Molecular Medicine, Cape Town, South Africa; 2 Infectious Diseases Epidemiology Unit, School of Public Health & Family Medicine, University of Cape Town, Cape Town, South Africa; 3 Department of Epidemiology, Mailman School of Public Health, Columbia University, New York, NY, USA

## Abstract

**Background:**

The impact of highly active antiretroviral therapy (HAART) on health-related quality of life has been widely researched in the developed world, but there are few data from sub-Saharan Africa, where the vast majority of HIV-infected individuals live. This study examined health-related quality of life among HIV-positive individuals initiating HAART in Cape Town, South Africa, and explored the impact of HAART-related drug toxicities on quality of life.

**Methods:**

Health-related quality of life was assessed using a standardised questionnaire, the Medical Outcomes Survey Short Form 36. Physical health summary scores and mental health summary scores were compared pre-HAART and at regular intervals during the first 48 weeks of HAART. The relationships between socio-demographic, baseline and on-treatment variables and decline in health-related quality of life, as well as the impact of drug toxicities on quality of life, were assessed in unadjusted bivariate and adjusted multivariate analyses.

**Results:**

Two hundred and ninety-five patients were enrolled into the study. There was a significant increase in health-related quality of life during the first 48 weeks on HAART. The median physical health summary score increased from 45 to 53 units (p < 0.001) and median mental health summary score increased from 45 to 50 units (p < 0.001).

The bulk of this increase occurred during the first 16 weeks. Overall, 23% of participants experienced a decline in their physical health summary score, while 34% showed a decline in the mental health summary score. Average drops in median physical and mental health summary scores were 8.4 units (SD 9.31) and 9.9 (SD 11.4) units respectively. Participants with drug toxicity had lower physical health summary scores than participants without drug toxicity at all time points. However, only three participants with toxicity (27%) reported an actual decline in health-related quality of life by week 48. Drug toxicities had little impact on mental health summary scores.

**Conclusion:**

These results confirm the health-related quality of life benefits of HAART. While the majority of patients experienced a significant improvement in health-related quality of life on HAART, up to a third of patients reported declines in this quality of life. This was largely related to better baseline clinical state. HAART-related drug toxicities did not have a significant impact on health-related quality of life during the first year of HAART, which supports the ongoing use of the current national first-line regimen.

## Background

By December 2006, an estimated 39.5 million people worldwide were living with HIV and a further 2.9 million people had died due to AIDS. The bulk of infections (63% of the global burden) occurred in sub-Saharan Africa, where 24.7 million people were reported to be HIV infected [1]. In South Africa alone, 5.4 million people were estimated to be infected with HIV by the middle of 2006 and 600 000 were thought to have AIDS [2].

Prior to 2004, people infected with HIV in South Africa who were unable to access life-saving antiretroviral (ARV) therapy progressed to AIDS and died of their disease. The rollout of highly active antiretroviral therapy (HAART) through national and provincial programmes has dramatically altered this experience.

By late 2008, an estimated 549 700 HIV-positive individuals were receiving HAART in South Africa [3]. With increasing numbers of HIV-positive individuals being enrolled onto HAART and increasing survival among these patients, there is a growing need to understand the impact of HAART use on the quality of lives of HIV-infected individuals [4-8].

There is a sizeable body of research on the impact of HAART on health-related quality of life (HRQoL) in the developed world. Most recent cohort studies in the USA and Europe have shown no significant change in HRQoL within the first two years of HAART [9-11], although one study showed an increase in mental quality of life only [12], and two showed a decrease in physical quality of life [13,14].

In contrast, the 2NN study, which compared the efficacy and safety of three non-nucleoside reverse transcriptase inhibitor (NNRTI)-containing regimens (nevirapine, efavirenz, and nevirapine plus efavirenz in combination with stavudine and lamivudine) showed an overall improvement in HRQoL over 48 weeks [15].

To date, only four studies have addressed the impact of HAART on HRQoL in developing countries [16-19]. Wouters et al [16] and Louwagie et al [17] assessed the impact of HAART on HRQoL in cross-sectional surveys, and showed a significant association between HAART and improved physical and emotional health. Unfortunately, the cross-sectional nature of these two studies and the limited time that participants were on HAART (six months or less) restrict the inferences that can be drawn from these studies.

Only the studies by Stangl et al [18] and Jelsma et al [19] assessed longitudinal changes in HRQoL associated with HAART use. Both reported a significant improvement in HRQoL over 12 months of HAART, with the bulk of this improvement occurring within the first three months on treatment.

Internationally, there is concern about the impact of HAART-related toxicity on HRQoL. In fact, it has been suggested that studies that consider only mortality outcomes ignore treatment-related morbidity and may actually overestimate the benefits of HAART [20].

These international concerns are echoed in South Africa and other developing countries, where national first-line regimens tend to be NNRTI-based and incorporate drugs such as stavudine, which has been shown to be the reason for up to 75% of drug switches for toxicity within the first three years of first-line HAART [21]. In South Africa, there is an ongoing debate about whether or not the side-effect profile of HAART may adversely affect the HRQoL of HIV-positive individuals.

More data about the impact of HAART and HAART-related toxicities on HRQoL are required in developing countries to inform programme and policy decisions about HAART roll-out strategies in order to maximise the quality of life of HIV-infected individuals.

## Methods

### Study population

This cohort study examined the HRQoL reported by HIV-positive individuals pre-HAART and at regular intervals during their first year of receiving HAART at the Hannan Crusaid Treatment Centre between September 2002 and March 2005. As per the national ARV guidelines, adult patients who had World Health Organization (WHO) Stage 4 disease and/or a CD4+ T cell count of <200 cells/mm^3 ^were commenced on first-line ARVs [22]. The majority of patients (99.6%) initiated on-treatment were ARV-naïve.

The Hannan Crusaid Treatment Centre is a community-based ARV clinic that was initiated in September 2002 as a joint venture between the Western Cape Department of Health, Desmond Tutu HIV Foundation and Crusaid, a UK-based non-governmental organization that raises funds to support people living with HIV/AIDS. The clinic was one of the first ARV rollout sites in the Western Cape Province of South Africa. It is situated alongside the primary community health care centre and boasts a multidisciplinary team of medical doctors, clinical nurse practitioners, clinic nurses, Sizophila adherence counsellors and a pharmacist.

The clinic followed a programmatic approach to ARV care with a standard first-line and second-line regimen. In keeping with WHO recommendations, the first-line regimen was NNRTI-based and the second was protease inhibitor-based [23].

Adults commenced on the first-line regimen (efavirenz or nevirapine plus stavudine and lamivudine) were reviewed at four, eight and 16 weeks, and thereafter every four months by a medical doctor. At these scheduled visits, patients were assessed clinically, virologically and immunologically. Patients who discontinued their first-line regimen – either due to virological failure or for toxicity reasons – were worked up for the second-line regimen (lopinavir/ritonavir, didanosine and zidovudine). Nucleoside reverse transcriptase inhibitor (NRTI) substitutions were made within regimen 1 or 2 for NRTI-associated toxicities.

### Study procedures

At the screening visit, HIV-positive individuals met with the clinic nurse, who completed a demographic information sheet. Blood was drawn for viral load, CD4 cell count and safety blood testing (including a full blood count and liver function tests) at the screening visit and at all subsequent scheduled visits prior to the patient seeing the medical doctor.

The adherence counsellors were trained in the administration of the HRQoL instrument. The quality of life questionnaire was administered at each of the following scheduled visits: screening, baseline, week 16, week 32, week 48 and week 64. Although HRQoL data continued to be collected at scheduled visits following week 64, this study focused on quality of life only during the first year of ARVs.

HRQoL data were intended to be collected on all patients at all scheduled visits within the first year. This, however, was not always possible. Reasons for incomplete HRQoL data were: death, loss to follow up, transfer out, and patients leaving the clinic without the questionnaire being administered. The analysis only included those patients with HRQoL data available at all time points during the first year on HAART.

The University of Cape Town Research Ethics Committee approved all research activities involving antiretroviral service delivery and patient outcomes at the site. Patients signed a research consent form at the screening visit, indicating their willingness to take part in this research study.

### Study measures

#### Quality of life

Health-related quality of life was assessed using a standardised questionnaire, the Medical Outcomes Survey Short Form 36 (MOS-SF36). The instrument uses 36 items to assess eight health concepts: (1) physical functioning; (2) role limitations because of physical health problems; (3) bodily pain; (4) social functioning; (5) general mental health; (6) role limitations because of emotional problems; (7) vitality; and (8) general health perceptions [24].

The MOS-SF36 questionnaire has been widely used in studies of quality of life in HIV-positive patients in both developed and developing countries, and has performed well in all of these settings [11,12,14,25-28]. The instrument has also undergone validity and reliability testing in a multiracial South African population and was able to differentiate between HIV-infected and non-infected individuals [28]. Population values exist for several countries, including South Africa. The English version of the instrument was used, with standard Xhosa explanations given by the counsellors for difficult concepts.

Quality of life data were entered using a custom-designed Epi Info™ template to ensure high data quality. On completion of data entry for each questionnaire, scores for the eight health components were automatically generated according to standard scoring algorithms. Data were then transferred into a Microsoft Excel spreadsheet where health component scores were transformed into the physical health summary (PHS) and mental health summary (MHS) scores using standardised factor analysis-based weights.

Missing questionnaire items were estimated using a standard scoring algorithm that estimates missing values [24]. Scores for screening and baseline were combined to form an average pre-HAART score. For those participants whose week 48 scores were not available, week 64 scores were used to replace missing data. This replacement of scores was deemed acceptable as data analysis demonstrated no significant difference between overall week 48 and week 64 scores.

#### Socio-demographic and clinical information

Demographic information was collected using standard paperwork. Patients were staged according to WHO clinical criteria by the medical doctor at their screening visit. The Toga Laboratory performed viral load and CD4 cell count testing. Viral load testing made use of the branch DNA hybridisation technique (Versant™ HIV-1 RNA 3.0 branched chain DNA assay, Bayer HealthCare, Leverkusen, Germany) and CD4+ T cell counts were measured by flow cytometry (FACSCount™, Becton Dickinson, Franklin Lakes, NJ, USA).

Drug toxicities were detected by the medical doctor at both scheduled and unscheduled clinical visits through clinical questioning, examination and safety blood draws (including a full blood count, liver function tests, amylase and lactate levels as requested). Drug toxicities were defined as any adverse event thought by the clinician to be HAART-related and that required a change in antiretroviral therapy. Drug changes could either be a NRTI substitution or a change from NNRTI to a protease inhibitor.

### Statistical analysis

The cohort was initially described using means, medians and proportions, as appropriate. Changes in HRQoL pre-HAART and at week 16, 32 and 48 were compared using the Wilcoxon Rank Sum Test. Crude associations were first examined using Fisher's Exact, Chi Squared and Wilcoxon Rank Sum tests, as appropriate.

Negative HRQoL was defined as a decrease in PHS or MHS scores between pre-HAART and week 48. Univariate relationships were then explored between the outcome variables – negative PHS and negative MHS – and each explanatory variable. Multivariate analyses made use of logistic regression models to examine the adjusted association between negative HRQoL and various socio-demographic, baseline and on-treatment explanatory variables as appropriate.

Multivariate analysis started with a full model (Model 1) and explanatory variables were removed in a stepwise manner until the final model (Model 2) was selected. All final logistic regression models were checked against model assumptions. Outliers and potentially influential observations were identified and examined to ensure that model results were not being unduly influenced by a small number of non-representative observations. Models were rerun with selected observations excluded.

All statistical analyses were performed using Intercooled Stata Version 8.2 (Stata Corporation, College Station, Texas, USA). All statistical tests are two-sided at alpha = 0.05.

## Results

Of the 295 patients with any HRQoL data, 292 (99%) had baseline data, 271 (92%) had week 16 data, 233 (79%) had week 32 data, and 179 (61%) had week 48 data. Complete HRQoL data, obtained pre-treatment and at every scheduled on-treatment visit, were available for 147 patients.

### Demographic, baseline and on-treatment characteristic

Table 1 describes the demographic and baseline characteristics of the 295 patients with any HRQoL data. The average age of the cohort was 34 years (standard deviation: 4) and 74% of patients were female (n = 219). Eighty-six percent of patients (n = 370) had WHO Stage 3 and 4 disease.

**Table 1:** Demographic, baseline and on-treatment characteristics of female and male patients with any health-related quality of life data

Variable	Total	Female	Male	P-value
Number	295	219	76	-

Age (years) (mean, (SD)	34 (7)	33 (7)	38 (6)	<0.001

WHO stage 3 & 4 (n,(%))	254 (86)	184 (86)	70 (92)	0.079

Baseline CD4 count cells/mm^3^ (median, (IQR))	88 (47; 148)	96 (52; 159)	77 (36; 130)	0.027

Baseline viral load copies/ml (median, (IQR))	80,876 (33,194; 201,784)	76,452 (31,547; 198,193)	87,763 (42,884; 211,938)	0.216

Baseline viral load log copies/ml (median, (IQR))	4.88 (4.52; 5.30)	4.88 (4.50; 5.30)	4.94 (4.63; 5.32)	0.216

Week 48 CD4 count cells/mm^3^ (median, (IQR))	261 (183; 340)	265 (205; 365)	215 (171; 304)	0.004

Week 48 viral load copies/ml (median,(IQR))	49 (49; 49)	49 (49; 49)	49 (49; 49)	0.340

Week 48 viral load log copies/ml (median,(IQR))	1.69 (1.69; 1.69)	1.69 (1.69; 1.69)	1.69 (1.69; 1.69)	0.276

Change in CD4 count cells/mm^3^ (mean, (SD))	178 (130)	184 (136)	159 (107)	0.152

Change in viral load log copies/ml (mean, (SD))	-2.96 (0.91)	-2.91 (0.95)	-3.11 (0.75)	0.107

Drug toxicity (n, (%))	11 (4%)	10 (5%)	1 (1%)	0.198

The median baseline CD4 count was 88 cells/mm^3 ^(inter quartile range: 44, 154) and median baseline log viral load was 4.9 (inter quartile range: 4.5, 5.3). Men were older and had more advanced disease than women. The majority of drug toxicities (90.9%) occurred in women with only one drug change made due to toxicity among men. There were no differences in demographic and baseline characteristics between patients with complete HRQoL data (n = 147) and those with incomplete data (n = 148).

### Health-related quality of life data

The median scores for the eight health concepts pre-treatment and at regular intervals on-treatment are described in Table 2. The scores all demonstrated an increase in HRQoL between pre-HAART and week 48, with the greatest increase occurring at week 16 (p < 0.001).

**Table 2:** Median scores for the eight health concepts pre-HAART and at week 16, 32 and 48 on HAART (n = 147)

	Pre-HAART	Week 16	Week 32	Week 48
Physical function	85	95	100	100

Physical role	50	100	100	100

Bodily pain	61	84	74	84

General health	54	77	77	72

Vitality	55	75	80	85

Social function	75	100	100	100

Emotional role	50	99	99	99

Mental health	68	72	76	72

**Figure 1:**
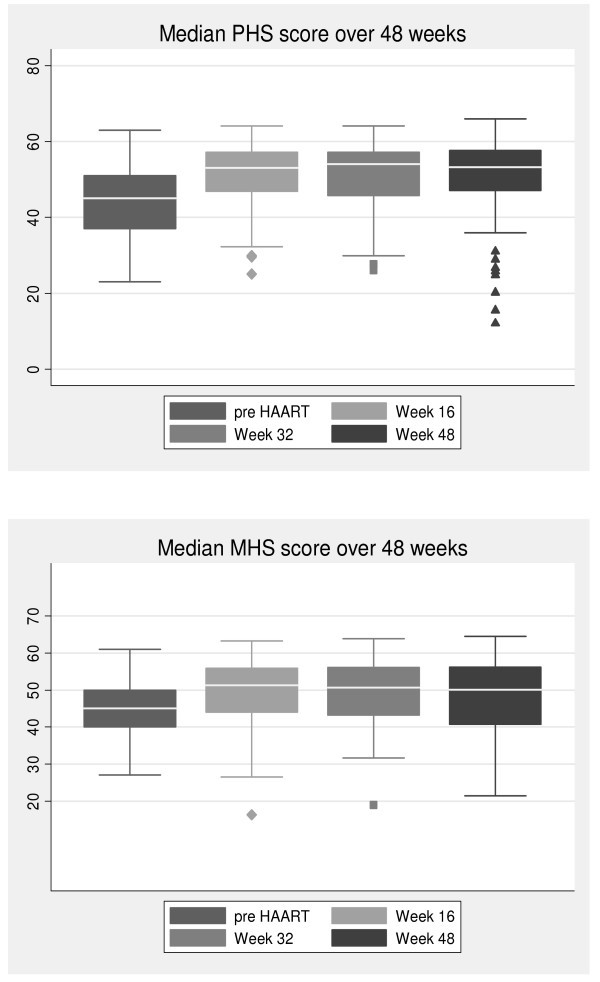
**Change in median physical health summary score and median mental health summary score over the first 48 weeks of HAART**.

The physical health summary and mental health summary scores also showed an improvement in HRQoL over time (Figure 1). There was a significant increase in both summary scores between pre-HAART and week 16. The median PHS score increased from 45 to 53 units (p < 0.001) and the median MHS score increased from 45 to 51 units (p < 0.001). These increases were then maintained through weeks 32 and 48.

**Figure 2:**
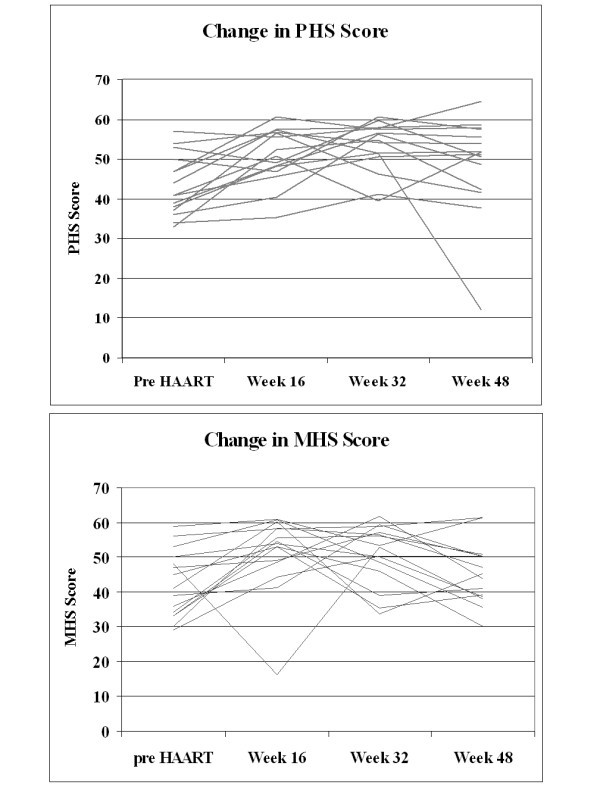
**Change in physical health summary score and mental health summary score over the first 48 weeks of HAART for 15 random participants**.

However, not all participants experienced a linear increase in HRQoL. Using a random sample of 15 participants, it was evident that while the bulk of participants experienced a gradual improvement in HRQoL, others experienced a worsening of HRQoL (Figure 2). While the average change in PHS score between pre-HAART and week 48 was an increase of seven units (standard deviation: 11.9), 23% of participants experienced a decrease in PHS score during this period. The average drop in PHS score among these participants was 8.4 units (standard deviation: 9.31).

Similarly, while MHS score increased by an average of 3.3 units (standard deviation: 11.4) between pre-HAART and week 48, 34% of participants experienced a decline in MHS score. The average drop in MHS score among these participants was 9.9 units (standard deviation: 5.92).

### Factors associated with negative change in quality of life

Baseline log viral load, pre-HAART PHS score, change in CD4 count and change in log viral load were all strongly associated with a negative PHS in the univariate analyses (Table 3).

**Table 3:** Factors associated with negative physical health summary score at week 48

Variable		Univariate model	Multivariate model 1	Multivariate model 2
		Odds ratio, 95% CI (P-value)	Odds ratio, 95% CI (P-value)	Odds ratio, 95% CI (P-value)

Age (continuous)		1.04, (0.98; 1.09) 0.184	1.08, (1.01; 1.15) 0.034	1.07 (1.01; 1.14) 0.029

Age (≤ 34 = 0, >34 = 1)		1.20, (0.56; 2.60) 0.637		

Gender (male = 0, female = 1)		0.55, (0.19; 1.56) 0.260	0.52, (0.14; 1.99) 0.341	

WHO stage (1&2 = 0, 3&4 = 1)		0.38, (0.13; 1.08) 0.068	0.61, (0.17; 2.15) 0.441	

Baseline CD4 count cells/mm^3^ (>50 = 0, ≤ 50 = 1)		0.42, (0.15; 1.17) 0.098	0.93, (0.25; 3.41) 0.910	

Baseline viral load log copies/ml (≤ 5 = 0, >5 = 1)		0.24, (0.10; 0.59) 0.002	0.48, (0.14; 1.65) 0.245	0.22, (0.08; 0.60) 0.003

Pre-HAART PHS score (continuous)		1.14 (1.07; 1.21) <0.001	1.16 (1.08; 1.24) <0.001	1.15 (1.08; 1.23) <0.001

Week 48 CD4 count cells/mm^3^ (<250 = 0, ≥ 250 = 1)		0.63, (0.29; 1.37) 0.245		

Week 48 CD4 count cells/mm^3^	<200	1		
	
	200–350	0.62, (0.25; 1.51) 0.29		
	
	350–500	0.67, (0.22; 2.07) 0.483		
	
	≥ 500	0.42, (0.08; 2.21) 0.309		

Week 48 viral load copies/ml (<50 = 0, ≥ 50 = 1)		0.96, (0.37; 2.48) 0.935		

Week 48 viral load copies/ml	<50	1		
	
	50–399	0.39, (0.08; 1.79) 0.224		
	
	400–4999	1.10, (0.11; 11.00) 0.936		
	
	≥ 5000	3.30, (0.77; 14.07) 0.107		

Week 48 log viral load log copies/ml (<1.69 = 0, ≥ 1.69 = 1)		0.96, (0.37; 2.48) 0.935		

Week 48 log viral load log copies/ml	<1.69	1		
	
	1.69–2.59	0.58, (0.16; 2.14) 0.414		
	
	2.60–3.69	1.10, (0.11; 11.00) 0.936		
	
	≥ 3.69	2.47, (0.51; 11.74) 0.225		

Δ CD4 count cells/mm^3^		0.995, (0.991; 0.999) 0.010	0.998, (0.993; 1.003) 0.420	

Δ log viral load log copies/ml		0.58, (0.37; 0.90) 0.015	0.66, (0.36; 1.27) 0.209	

In the multivariate regression model, pre-HAART PHS score and baseline log viral load were the strongest predictors of negative PHS at week 48. Participants with a higher pre-HAART HRQoL score were more likely to report negative PHS (OR1.15; 95% CI 1.08, 1.23; p < 0.001), whereas participants with a higher baseline log viral load (>5.0 log) were less likely to report negative PHS than participants with a lower baseline log viral load (≤ 5.0 log) (OR 0.22; 95% CI 0.08, 0.60; p = 0.003).

Age was also predictive of negative PHS. Older participants (above 34 years of age) were more likely to report negative PHS than younger participants (OR 1.07; 95% CI 1.01, 1.14, 0.085; p = 0.029). Neither gender nor any of the week 48 variables were associated with negative PHS.

Baseline CD4 count, baseline log viral load, pre-HAART MHS score and change in log viral load were all associated with negative MHS in the univariate models (Table 4). In the multivariate regression model, pre-HAART MHS score was the strongest predictor of negative MHS at 48 weeks. Participants with higher pre-HAART HRQoL scores were more likely to report negative MHS than participants with lower pre-HAART MHS scores (OR 1.09; 95% CI 1.04, 1.15; p = 0.001).

**Table 4:** Factors associated with negative mental health summary score at week 48

Variable		Univariate model	Multivariate model 1	Multivariate model 2
		**Odds ratio, 95% CI** **(P-value)**	**Odds ratio, 95% CI** **(P-value)**	**Odds ratio, 95% CI** **(P-value)**

Age (continuous)		1.01, (0.96; 1.06) 0.767		

Age (<34 = 0, >34 = 1)		1.49, (0.75; 2.96) 0.258	2.20, (0.94; 5.15) 0.068	1.77, (0.83; 3.78) 0.142

Gender (male = 0, female = 1)		0.58, (0.24; 1.41) 0.227	0.65, (0.23; 1.85) 0.419	

WHO stage (1&2 = 0, 3&4 = 1)		0.94, (0.33; 2.71) 0.906	1.45, (0.44; 4.83) 0.543	

Baseline CD4 count cells/mm^3^ (>50 = 0, <50 = 1)		0.35, (0.14; 0.86) 0.022	0.38, (0.14; 1.07) 0.067	0.41, (0.16; 1.09) 0.075

Baseline viral load log copies/ml (<5 = 0, >5 = 1)		0.41, (0.20; 0.83) 0.014	0.55, (0.20; 1.49) 0.239	0.50, (0.23; 1.09) 0.081

Pre-HAART MHS score (continuous)		1.10 (1.05; 1.16) <0.001	1.10 (1.04; 1.16) <0.001	1.09 (1.04; 1.15) 0.001

Week 48 CD4 count cells/mm^3^ (<250 = 0, >250 = 1)		1.67, (0.83; 3.37) 0.151		

Week 48 CD4 count cells/mm^3^	<200	1		
	
	200–350	5.18, (1.92; 13.96) 0.001		
	
	350–500	2.39, (0.72; 7.91) 0.155		
	
	>500	2.52, (0.58; 10.88) 0.216		

Week 48 viral load copies/ml (<50 = 0, >50 = 1)		1.30, (0.57; 2.94) 0.535		

Week 48 viral load copies/ml	<50	1		
	
	50–399	0.95, (0.33; 2.69) 0.919		
	
	400–4999	2.05, (0.28; 15.14) 0.481		
	
	>5000	2.05, (0.49; 8.66) 0.327		

Week 48 viral load log copies/ml (<1.69 = 0, >1.69 = 1)		1.30, (0.57; 2.94) 0.535		

Week 48 viral load log copies/ml	<1.69	1		
	
	1.69–2.59	0.88, (0.31; 2.47) 0.808		
	
	2.60–3.69	2.05, (0.28; 15.14) 0.481		
	
	>3.69	2.74, (0.58; 12.85) 0.202		

Δ CD4 count cells/mm^3^		0.999, (0.996; 1.002) 0.414	1.002, (0.998; 1.01) 0.389	

Δ log viral load log copies/ml		0.67, (0.44; 1.01) 0.054	0.74, (0.43; 1.28) 0.282	

Baseline CD4 count and baseline log viral load remained weakly associated with the outcome. Participants with lower baseline CD4 count (≤ 50 cells/mm^3^) were less likely to experience negative MHS than participants with higher baseline CD4 count (>50 cells/mm^3^) (OR 0.41; 95% CI 0.16, 1.09; p = 0.075). Participants with a higher baseline log viral load (>5.0 log) were less likely to experience negative MHS than participants with lower baseline log viral load (≤ 5.0 log) (OR 0.50; 95% CI 0.23, 1.09; p = 0.081). Gender and the week 48 variables were not predictive of negative MHS at 48 weeks.

### Drug toxicities and quality of life

Eleven participants experienced drug-related toxicities during the first 48 weeks of HAART. Ninety-one percent (n = 10) of these toxicities occurred in women, with 50% (n = 5) of these being due to lactic acidosis. Participants experiencing drug toxicities had similar demographic and baseline characteristics to the overall cohort.

Table 5 describes the types of drug toxicities that occurred during the first year of HAART. Efavirenz hypersensitivity reactions were the cause of drug toxicities within the first 16 weeks of HAART. Between weeks 16 and 32, peripheral neuropathies were the main reason for drug changes. Elevated transaminases and hyperlactatemia were the main causes of drug toxicities between weeks 32 and 48 of HAART.

**Table 5:** Description of drug toxicities occurring during the first year of HAART

Description	Week 0–16	Week 16–32	Week 32–48	Total
Any toxicity	2	2	7	11

EFV hypersensitivity reaction	2	***	1	3

Peripheral neuropathy	***	2	***	2

Elevated transaminases	***	***	1	1

Hyperlactataemia/lactic acidosis	***	***	5	5

One patient experienced an efavirenz hypersensitivity reaction between weeks 32 and 48. This was due to the fact that the patient was switched to efavirenz at this time. The bulk (64%) of toxicities occurred during the week 32 to 48 interval and were mostly elevated transaminases and hyperlactataemia related to stavudine use.

The 11 participants who experienced drug toxicity during the first 48 weeks of HAART achieved lower PHS scores at all time points than the 281 participants who did not have toxicity. While these differences were not statistically significant pre-HAART and at weeks 16 and 32, they did become significant at week 48. The median PHS score at week 48 was 50 for participants with drug toxicity, compared to 53 for participants without drug toxicity (p = 0.0053) (Table 6).

**Table 6:** Median physical health summary and median mental health summary scores and drug toxicity

	N	Pre-HAART	Week 16	Week 32	Week 48
**Median PHS**					

Drug toxicity	11	39	51	52	50

No drug toxicity	281	44	52	53	53

p-value		0.854	0.225	0.390	0.005

**Median MHS**					

Drug toxicity	11	42	51	51	52

No drug toxicity	281	45	50	49	48

p-value		0.179	0.539	0.326	0.912

Median PHS and median MHS pre-HAART and at week 16, 32 and 48 in patients with any drug toxicity at any time in the first year compared to patients without drug toxicity.

Drug toxicities did not appear to have a significant impact on median MHS scores over the first 48 weeks of HAART. The 11 participants with drug toxicity had a lower median MHS score pre-HAART than the 281 participants without drug toxicity, but this difference was not statistically significant (42 versus 45, p = 0.1793). There was no impact of baseline mental health status on the reporting of toxicities. At weeks 16, 32 and 48, participants with drug toxicity reported higher median MHS scores than those without toxicity. Again, these differences were not statistically significant (Table 6).

Examining the associations between drug toxicity and negative HRQoL, it was noted that only three (27%) of all drug toxicities occurred among participants who reported negative PHS and that these toxicities occurred during the week 32 to 48 treatment interval. No drug toxicities occurred among participants who reported negative MHS.

## Discussion

This study reported a significant increase in HRQoL during the first 48 weeks on HAART, with the bulk of this increase occurring during the first 16 weeks on treatment. Improvement in HRQoL occurred across all core domains assessed, as well as the physical health summary and mental health summary.

This study therefore supports the findings of Stangl et al [18] and Jelsma et al [19] who both reported an increase in HRQoL within the first three months of therapy in similar patient populations. The dramatic increase in HRQoL during the first few weeks of HAART occurred over the time period when patients usually experience the most significant gains in health. The greatest decrease in viral load happens within the first few weeks of treatment and mortality and morbidity rates begin to fall after just a month on HAART [29,30].

There have been few analyses dealing with declines in PHS and MHS scores. In fact, negative HRQoL is often overshadowed by the overwhelming positive impact of HAART on HRQoL, and is therefore not reported. This research showed that although there was a general improvement in HRQoL on HAART, up to a third of participants experienced a decline in HRQoL during the first 48 weeks of HAART. Twenty-three percent of participants reported a drop in PHS score and 34% reported a drop in MHS score.

The most significant predictors of negative PHS and MHS were baseline HRQoL score, baseline log viral load and baseline CD4 count. The association between higher baseline HRQoL score and negative HRQoL could have been due to the fact that patients with higher baseline scores had less room for improvement and were therefore more likely to regress to the mean.

Baseline log viral load was strongly associated with negative PHS. Participants with higher baseline log viral loads were less likely to report negative PHS than participants with lower baseline log viral loads. Similarly, baseline log viral load and baseline CD4 count were associated with negative MHS. Participants with higher baseline log viral loads or lower baseline CD4 counts were less likely to report negative MHS than participants with lower baseline log viral loads or higher baseline CD4 counts.

Participants with more advanced disease, characterised by higher baseline viral loads and lower baseline CD4 counts, were less likely to report a decline in HRQoL than those with earlier disease. So it was the relatively well patients entering into the programme who were at greatest risk of experiencing negative HRQoL. These associations could be explained by the negative impact of symptoms on HRQoL [10-12]. Patients with more advanced disease are more likely to have a greater number or intensity of symptoms than patients with less advanced disease pre-HAART [10], but once on HAART, these symptoms improve [11,12].

Although there is great concern about the impact of drug toxicities on HRQoL, few studies have directly assessed this association. In the developed world, symptoms have been shown to impact negatively on both physical and mental HRQoL [10,31]. However, the nature of the symptoms and whether they were attributed to the disease process or to HAART was not clear.

In the developing world, Jelsma et al [19] concluded that possible side effects of HAART had a negligible impact on HRQoL. This conclusion was based on the overall increase in HRQoL demonstrated for the cohort and did not specifically address those patients who experienced a decline in HRQoL.

Few drug toxicities were recorded during the first 48 weeks on HAART. While participants who experienced drug toxicity had lower PHS scores than participants without a drug toxicity at all time points (most notably during the 32 to 48 week treatment interval), only three (27%) participants with toxicity reported an actual decline in physical HRQoL between pre-HAART and week 48.

Drug toxicities, especially those related to stavudine use, may have a negative impact on physical HRQoL at the time of the toxicity. They do not, however, reduce overall gains in HRQoL. Drug toxicities had little impact on mental HRQoL.

The greatest strength of this study is its longitudinal design. This allowed the assessment of the associations between various socio-demographic and clinical factors and HRQoL, and allowed for inferences to be made about causal relationships.

Limitations to the study included possible selection bias and information bias related to the use of the MOS-SF36 instrument. The loss of up to 50% of patients, who had incomplete HRQoL data, from the final analysis may have resulted in a selection bias towards the healthier section of the cohort. However, as there were no significant differences in demographic, baseline and on-treatment characteristics between patients with incomplete and complete HRQoL data, it is unlikely that this was the case. This did impact on the number of patients available for analysis though, and may have limited the ability of the study to detect significant associations.

The MOS-SF36 instrument is a generic HRQoL measure and, like all generic instruments, may not be sensitive enough to measure the more specific aspects of HRQoL impacted on by the HIV disease process. This could lead to either an underestimation or overestimation of HRQoL scores and, more important, to a change in HRQoL scores, thereby under-reporting or over-reporting the actual impact of HAART on HRQoL.

The MOS-SF36 instrument is also prone to ceiling effects, where substantial numbers of patients get the highest possible score for a domain. This would have made it difficult for the instrument to pick up changes in HRQoL at the upper end of the scale, and may have been a problem with increasing time on treatment. Ceiling effects could have lead to the underestimation of HRQoL gains.

This study reported on HRQoL during the first 48 weeks of HAART only. As HAART-related drug toxicities, especially those related to stavudine, increase with length of time on HAART [21], this study may have under-reported the impact of drug toxicities on gains in HRQoL.

Furthermore, this study only considered drug toxicities that were severe enough to prompt a change in antiretroviral therapy. Less severe toxicities that may also have impacted negatively on HRQoL were not reported on. This could have led to an underestimation of the true negative impact of drug toxicities on HRQoL.

## Conclusion

This study confirmed the overwhelmingly positive HRQoL benefits of HAART in a community ARV clinic in South Africa. HRQoL improved significantly during the first 48 weeks of HAART, with the bulk of improvement occurring during the first 16 weeks of treatment.

Up to a third of patients experienced a decline in HRQoL on HAART. This was largely related to the patient's baseline clinical state. HAART-related drug toxicities did not have a significant impact on HRQoL during the first year of HAART, supporting the ongoing use of the current national first-line regimen.

## Competing interests

The authors declare that they have no competing interests.

## Authors' contributions

JP participated in the design of the study and its coordination, acquired the data, performed the statistical analysis, interpreted the data, and drafted the manuscript. LM assisted with the statistical analysis and interpretation of the data, and helped to draft the manuscript. RW conceived of the study, participated in its design, and critically reviewed the manuscript. All authors have read and approved the final manuscript.
